# Modern Trends in Natural Antibiotic Discovery

**DOI:** 10.3390/life13051073

**Published:** 2023-04-23

**Authors:** Anna A. Baranova, Vera A. Alferova, Vladimir A. Korshun, Anton P. Tyurin

**Affiliations:** 1 Shemyakin-Ovchinnikov Institute of Bioorganic Chemistry, Miklukho-Maklaya 16/10, 117997 Moscow, Russia; anjabaranowa@list.ru (A.A.B.); alferovava@gmail.com (V.A.A.); 2 Gause Institute of New Antibiotics, Bolshaya Pirogovskaya 11, 119021 Moscow, Russia

**Keywords:** antibiotics, natural products, dereplication, BGC activation, genome mining, in situ cultivation, co-cultivation

## Abstract

Natural scaffolds remain an important basis for drug development. Therefore, approaches to natural bioactive compound discovery attract significant attention. In this account, we summarize modern and emerging trends in the screening and identification of natural antibiotics. The methods are divided into three large groups: approaches based on microbiology, chemistry, and molecular biology. The scientific potential of the methods is illustrated with the most prominent and recent results.

## 1. Introduction

Prevention and treatment of many infectious diseases is impossible without the use of antibiotics. At the same time, the efficacy of medical antibiotics is steadily declining due to the spread of antimicrobial resistance on the one hand, and the lack of fundamentally new antimicrobial agents on the other. The WHO already lists some infections caused by resistant bacteria as a “critical priority” for the development of new antibiotics [1].

Selman Waksman, one of the pioneers in the development of medical antibiotics, defined antibiotics as “a chemical substance, produced by micro-organisms, which has the capacity to inhibit the growth of and even to destroy bacteria and other micro-organisms” [2]. Since then, the definition has undergone many changes [3,4,5], and there is still no consensus. Today, “antibiotics” usually refers to antibacterial therapeutic agents based on small molecules, without insisting on their production by microorganisms. Therefore, “antibiotics” can be natural products, their semisynthetic derivatives, or fully synthetic substances. Each group contributes to the fight against microbial infections. Semisynthetic antibiotics rely on parent natural precursors, and fully synthetic antibacterials are often bioinspired. Therefore, broadly speaking, natural compounds are the main source of new antimicrobial agents [6,7].

**Figure 1:**
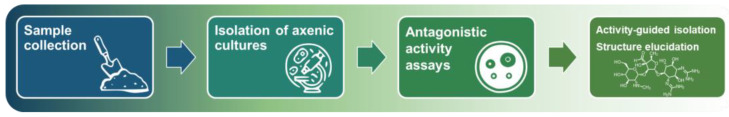
Traditional phenotypic screening (the “Waksman platform”).

Together with the definition of antibiotics, Waksman introduced the first platform for systematic screening of new antibiotics [8]. The workflow includes the isolation of soil-dwelling microorganisms, largely actinobacteria, and a growth inhibition assay. The subsequent purification of active compounds from selected culture broths gave us many useful natural products (Figure 1). The main classes of medicinal antibiotics were discovered in this “golden age”—1940–1960s—when the Waksman phenotypic screening approach was exploited by many groups both in academia and in the pharmaceutical industry [9]. The stagnation in antibiotic discovery, along with various difficulties in their development, allows one to ask a question: “Is the success story over?” [10]. Due to a variety of issues, antibiotic development became very risky and costly for pharmaceutical companies. Since improving antibiotic development requires primarily legislative solutions, there is little that researchers can contribute. However, scientists can and should extract all useful antimicrobial substances from nature to the largest extent possible. Thus, let us take a look at the current trends in this area.

After decades of success, researchers recognized the limitations of the “Waksman platform”:

1. Most of the soil microbiome is unculturable in standard lab conditions. Even for actinomycetes, classical isolation methods yield a large number of Streptomyces colonies, but other species of the class are underrepresented. As a result, we cannot evaluate the biosynthetic potential of the majority of microorganisms (the microbial dark matter problem).
2. Screening of cultures with broad antimicrobial activity often yields toxic and/or well-known compounds (the re-isolation problem).
3. The screening requires prolonged cultivations (to isolate axenic cultures, for test fermentations, etc.) and resource-consuming activity-guided isolation of antibiotics. In general, it cannot be adapted for fast and high-throughput screening.

**Figure 2:**
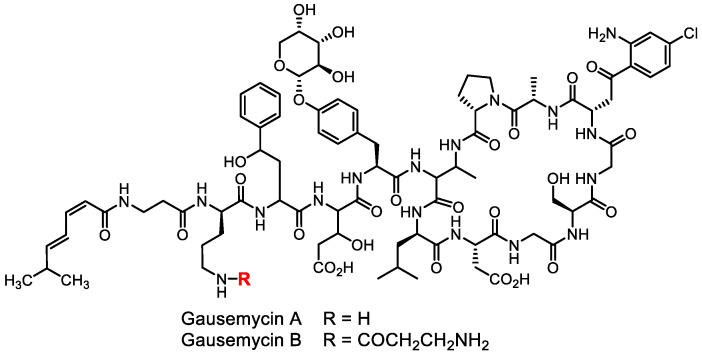
Structures of gausemycins, recently discovered lipoglycopeptide antibiotics.

Although random search sporadically provides some interesting results (e.g., the recent discovery of gausemycins (Figure 2), a new class of lipoglycopeptide antibiotics possessing an original mechanism of action against Gram-positive bacteria [11]), it cannot be considered a modern methodology anymore.

**Figure 3:**
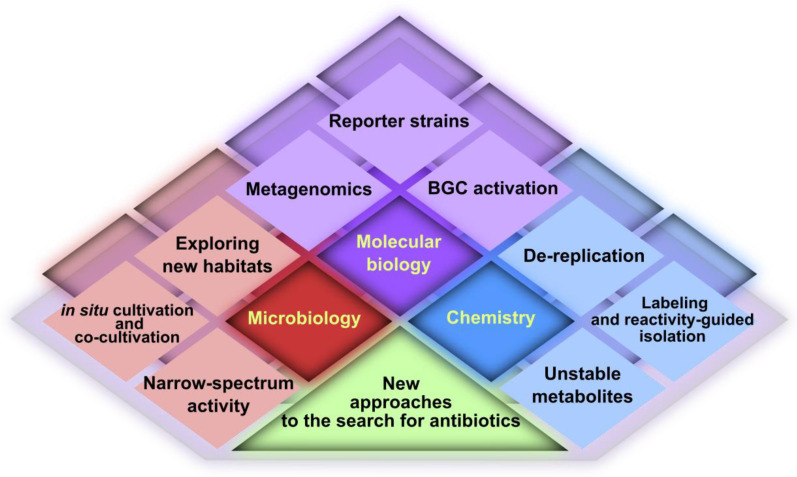
Main modern approaches in the screening of new antibiotics from natural sources.

In this review, we summarize the main advancements that overcome the limitations of traditional phenotypic screening or increase its efficiency. We have critically analyzed both the methodology and the outcome of recent studies (from 2012 to the present). The new approaches emerging in this multidisciplinary area of research can be schematically divided into three main classes, as shown in Figure 3.

## 2. Microbiology

Isolating antibiotic producers the traditional way leads to systematic selection of only a small fraction of the existing microbial diversity. New genomic data [12] indicate that the production of specific metabolites can occur in uncultured microbial taxa. Obviously, only a small portion of the antibiotics produced by microorganisms has been selected so far, while their biosynthetic potential is much higher [13].

**Figure 4:**
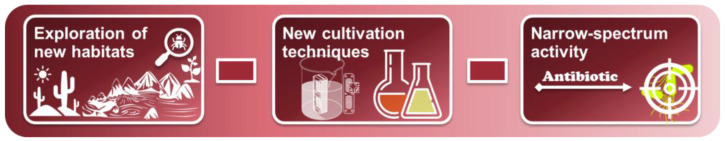
Basic microbiological approaches to the search for new antibiotics.

Novel data spurred ecology-driven antibiotic discovery in understudied environments (Figure 4). Since the adaptation of microorganisms to specific ecological niches is realized by bizarre biochemistry and unusual pathways and metabolism, such microorganisms can be a source of new biosynthetic gene clusters [14]. Moreover, even for well-studied habitats, it is possible to increase the seeding capacity of the producers. It is known that 95–99% of the total microbiome belongs to microorganisms that are not cultivatable under standard laboratory conditions [15,16,17,18]. New cultivation technologies enable access to a part of this cryptic microbiome.

Another step at which some potentially valuable antibiotic metabolites are lost is primary screening. Most often, producers exhibiting a broad spectrum of activity of cultural broth are selected. Recently, the paradigm has changed, and metabolites with more selective action are attracting more and more attention [19].

### 2.1. Exploring New Habitats

The high frequency of streptomycetes and the rediscovery problem have led to a natural decline in interest in soil microorganisms. Since most antibacterial agents come from well-known terrestrial or aquatic actinomycete isolates [20], researchers focused on microorganisms isolated from unusual habitats [21]. The search for new bioactive molecules has shifted toward marine ecosystems [22,23,24], taxa living in extreme environments [25,26,27,28], plant-associated endophytes [29,30] and epiphytes [31], and hard-to-reach habitats (such as karst caves) [32].

Marine ecosystems are highly diverse in terms of temperature fluctuations, pressure, light, composition and nutrient concentration. Due to the unique features of polyextremophilic adaptation and a wide range of secondary metabolites, producers of marine origin are an interesting subject in terms of expanding the space of pharmacophores [28,33,34]. Microorganisms of marine origin are often associated with various marine invertebrates, algae and plants, contributing to the nutrient cycle and decomposition of organic matter [35]. Therefore, they produce a huge number of secondary metabolites with antitumor and antibiotic activity [28,36]. The main source of the majority of modern antibiotics of natural origin is actinobacteria, which can live in a variety of environmental conditions [37,38,39,40].

Natural products produced in extreme environments are optimized for these niches and might require significant modification to work in humans. Certain symbiont habitats impose requirements on microorganisms that facilitate the selection of metabolites with high therapeutic potential (e.g., nontoxic to mammals and active against Gram-negative bacteria) [14]. Bacteria and fungi that participate in symbiosis are an area that still remains underexplored for the discovery and development of new antibiotics. Symbiosis with plants, invertebrates, marine sponges and other organisms has evolved under the influence of the host organism, which makes bacterial symbionts a promising source of unusual metabolites [41,42,43].

**Figure 5:**
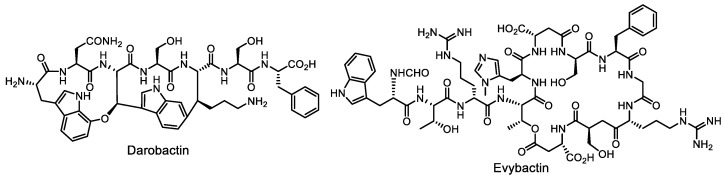
Peptide antibiotics darobactin and evybactin, recently found to be produced by the nematode microbiome.

One such microorganism is *Photorhabdus* sp., a nematophilic bacteria, a key member of the nematode gut microbiome. Darobactin (Figure 5), an antibiotic isolated in 2019 by a group led by Kim Lewis, was derived from the nematode symbiont strain *Photorhabdus khanii* HGB1456 [44]. Darobactin acts against Gram-negative bacteria by inhibiting the assembly of outer membrane proteins [45]. In 2022, the Lewis group isolated a new potent and selective antibiotic, evybactin (Figure 5), active against *Mycobacterium tuberculosis*, from another nematode symbiont strain, *Photorhabdus noenieputensis* DSM 25462 [46].

Endophytic microorganisms, especially actinomycetes [47,48] and fungi [49], possess a great variety of biologically active metabolites. Endophytic bacteria are used not only in medicine and veterinary medicine, but also as biofertilizers and biocontrol agents in agriculture [50,51].

### 2.2. New Cultivation Techniques

#### 2.2.1. The Co-Cultivation Approach

The complete diversity of secondary metabolites produced by microorganisms cannot be harvested by standard monocultivation techniques. Unconventional methods are needed to awaken “silent” biosynthetic pathways and induce the production of secondary metabolites. One of the most promising approaches to in vitro cultivation is simulation of the natural interaction between different species of microorganisms by means of co-culturing [52] to obtain a large variety of secondary metabolites [53]. In a co-culture, two or more distinct microorganisms are incubated together in order to mimic the natural environment [52,54,55]. It is possible that this method of cultivation will contribute to the disinhibition of the “silent” genes responsible for the production of certain antibiotics. Moreover, co-cultivation may lead to diversification of secondary metabolites by biotransformation by other species.

The interest in obtaining secondary metabolites by co-culturing a fungus with a fungus is also increasing every year [56]. Interestingly, the most common microorganisms that co-ferments with other microbes and produces numerous new chemical structures are fungi of the genus *Aspergillus* [57].

#### 2.2.2. In Situ Cultivation

As mentioned above, most species of microorganisms are unculturable and cannot grow under normal laboratory conditions [58], and alternative methods of in situ cultivation are being considered for them: diffusion chambers [59], iChip [60], microbial traps [61], the double encapsulation technique [62] and others. Each of these methods leads to an increase in microorganism isolation rate [58]. For example, three times as many new bacterial species were isolated using a diffusion chamber compared to standard direct plating cultivation [63].

**Figure 6:**
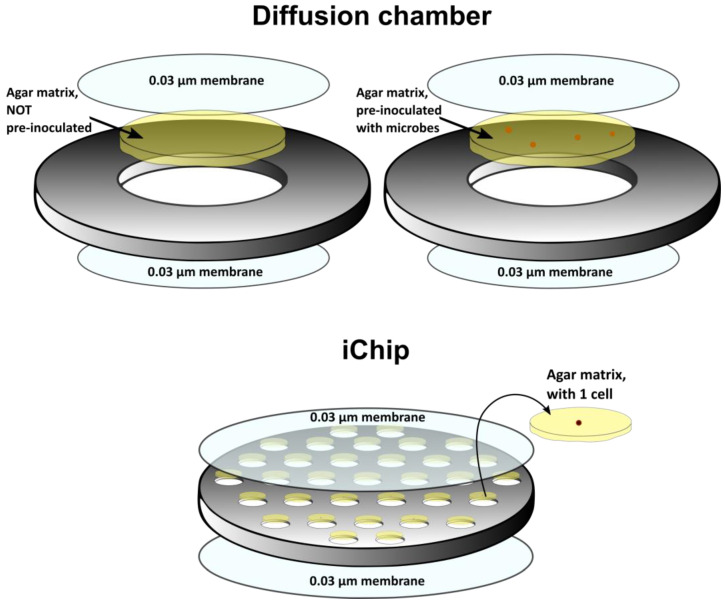
Instrumentation of the iChip technology: diffusion chambers and their multiplication on iChip.

The diffusion chamber consists of a stainless steel or plastic washer and membranes with a pore size of 0.03 µm (Figure 6). After attaching the membrane on one side, the inoculum is placed inside the chamber, and then the chamber is closed on the reverse side with another membrane. After assembly, the chamber is placed in the initial natural medium, and the inoculated microbes are incubated for several weeks. During in situ cultivation, the membrane ensures the exchange of growth factors and nutrients between the natural environment and the agar inside the chamber. This approach should minimize the differences in the chemical environment on both sides of the membrane, thus mimicking natural conditions inside the chamber [59].

The essence of the isolation chip technology (iChip), a high-throughput cultivation method, is the use of a diffusion chamber-type device with a small cell size, closed on both sides by a fine-pored track membrane. The polycarbonate membrane allows only low-molecular-weight substances to pass [64].

**Figure 7:**
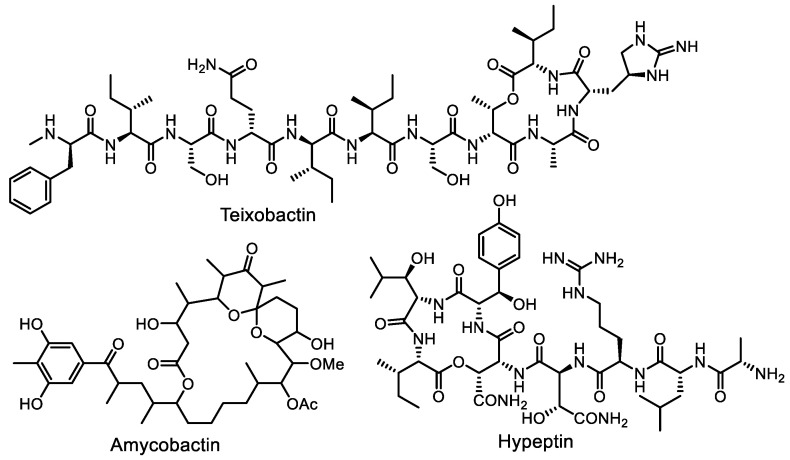
Antibiotics teixobactin, amycobactin, and hypeptin, recently discovered using the iChip technology.

This technology made it possible to isolate the beta-proteobacterium *Eleftheria terrae*, a producer of the antibiotic teixobactin (Figure 7), a non-ribosomal peptide with an original structure, from soil [65]. Teixobactin inhibits both peptidoglycan and teichoic acid synthesis by binding bactoprenol-coupled cell wall precursors [66]. The macrolide amicobactin, which showed antituberculosis activity, was obtained this way [67]. Hypeptin, an antibiotic produced by *Lysobacter* sp. K5869 and obtained using the iChip technology, has common structural features with teixobactin and exhibits potent activity against a wide range of Gram-positive bacteria [68].

#### 2.2.3. Microtechnology

Various microtechnologies can also be used to increase microbial biodiversity. They allow to improve of high-throughput screening, reducing the time and resources needed for experiments. Recently [69], three main microtechnology methods have appeared: Microarrays, microencapsulation [70] and micromechanical devices and microfluidics [71].

Microfluidics, a technique that has been rapidly developing in recent years, is worth mentioning in more detail [72]. This droplet-based technique generates homogeneous microdroplets under precise control at the picoliter or nanoliter scale upon high-frequency vibration (~kHz) [73]. The droplets can function as bioreactors for versatile chemical/biological studies. Taking advantage of a discrete compartment with limited volume, single-cell isolation and manipulation, improved effective concentrations in droplets, elimination of heterogeneous population effects and reduced contamination risks, this technique is a powerful tool for rapid, sensitive and high-throughput detection and analysis of bacteria, even for rare or unculturable strains [74,75,76]. For example, a platform for ultra-high-throughput screening in microfluidic double water-in-oil-in-water emulsion droplets was created [77]. The method is based on the encapsulation of microorganisms into droplets of a monodisperse microfluidic double water-in-oil-in-water emulsion (MDE) and fluorescence-activated cell sorters (FACS) [71,78,79]. The MDE-FACS platform is applicable to a variety of activity types and in-depth microbial community profiling.

Despite the success in the technical implementation of microfluidic cultivation into the antibiotic screening process [80,81,82,83], no new scaffolds have been discovered using this technology.

### 2.3. New Approaches to Phenotypic Screening (Narrow-Spectrum Activity)

The use of broad-spectrum antibiotics has many side effects: it affects the stability of the human microbiome and the resistance of unrelated pathogens. This encourages researchers to search for drugs with a more selective effect [84] among natural antibiotics [85].

**Figure 8:**
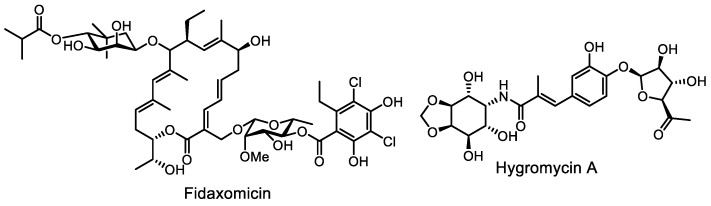
Repurposed antibiotics fidaxomicin and hygromycin A.

The key idea of the approach to finding new antibiotics with a narrow spectrum of action was proposed back in 2016 by Brown and Wright [19]. The idea was that, in high-throughput screening, one should select cultures and substances with a narrow spectrum of action or repurpose already known antibiotics. This idea is largely inspired by the example of the natural antibiotic fidaxomicin, introduced into clinical practice in 2010 as a bactericidal antibiotic of narrow action against Gram-positive anaerobic bacteria, primarily *Clostridium difficile* (the main cause of hospital mortality, affecting the human gut and causing fatal diarrhea) [86]. Fidaxomicin (Figure 8) selectively inhibits the *Clostridium difficile* RNA polymerase with minimal effects on gut commensals, such as Proteobacteria and Bacteroidetes [87].

An example of a successful implementation of this approach is hygromycin A (Figure 8), produced by the actinomycete *Streptomyces hygroscopicus*. This known substance showed highly selective activity against spirochetes, including *Borrelia burgdorferi*. Unexpectedly, it was found that this compound inhibited the growth of *B. burgdorferi* and did not affect the gut microbiome, unlike clinically relevant antibiotics. The compound was tested in a mouse model of acute Lyme disease and showed efficacy when administered both intraperitoneally and orally. This selective antibiotic could, in the future, provide better therapy for Lyme disease and eradicate it from the environment [88].

**Figure 9:**
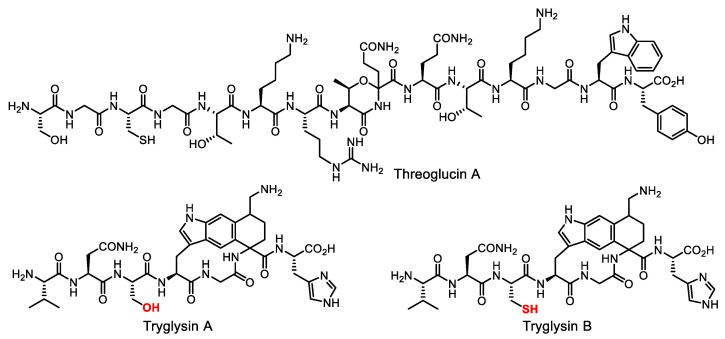
Narrow-spectrum antibiotics threoglucin A and tryglusins A and B.

This approach opens up prospects for the study of bacteriocins and related strain-specific agents as next-generation medicines. For example, threoglucin A (Figure 9), a post-translationally modified peptide, had remarkable narrow-spectrum activity: a bacteriostatic effect had been detected only for *Streptococcus suis*. Coupled with low toxicity to human cells in vitro, these properties make threoglucins interesting as drug leads [89]. Similarly, tryglysins (Figure 9) specifically inhibit the growth of several streptococcal strains, but not of other Gram-positive bacteria [90].

Thus, the search for narrow-spectrum antibiotics can both help identify unusual structures in primary screening and find new applications for previously described compounds. Treatment with narrow-spectrum antibiotics reduces the rate of spread of antibiotic resistance and reduces unwanted side effects of antibiotic therapy [91]. It should be noted that funding agencies tend to prefer to support the development of broad-spectrum drugs over more selective candidates. Therefore, the introduction of narrow-spectrum antibiotics into clinical practice is hindered.

## 3. Molecular Biology

**Figure 10:**
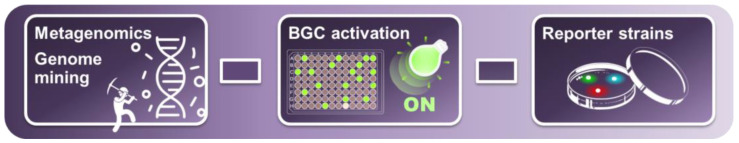
Basic molecular biology approaches to the search for new antibiotics.

Natural antibiotics are products of biosynthesis. This transformation of simple precursors into complex compounds is encoded in the genome, usually as a biosynthetic gene cluster (BGC). In this section, we have grouped together various approaches based on processing genomic data to find and activate of BGCs (Figure 10): the first part primarily collects methods based on homo/heterologous expression, and the second one contains methods for activating the clusters in a native producer. In the third part, we have included new methods for mechanism-based screening, since they are impossible without appropriate methods for genetic engineering, however, applied not to producers, but to test cultures.

### 3.1. Metagenomic Screening and Genome Mining

The search for biosynthetic gene clusters in metagenomic data and their subsequent heterologous expression theoretically opens up the entire biosynthetic potential of microorganisms [92]. Developments in next-generation sequencing technologies have brought the recognition of microbial genomes as a rich resource for novel natural product discovery. The development of community resources for the integration of genomic and metabolomic data spurs interest in this approach [93,94,95]. Genomic studies show high cryptic biotechnological potential even in actively cultured and studied actinobacteria [96]. The study of microorganisms from underexploited ecological niches is of the most interest [97]. There is also considerable interest in metagenomic studies of the human microbiota as a source of antibiotic compounds [95,98,99]. Recently, several approaches to the search for new antibiotics of various biosynthetic origins, nonribosomal peptides, ribosomally synthesized and post-translationally modified peptides (RiPPs) and polyketides have been successfully applied.

**Figure 11:**
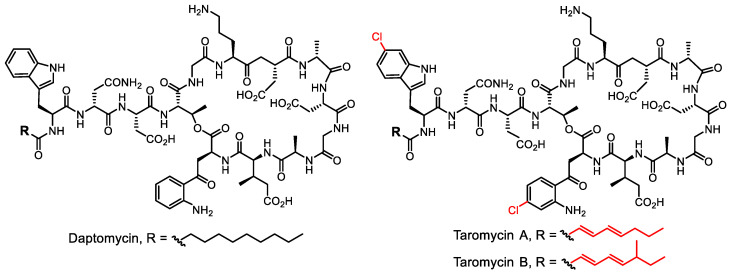
Clinically approved antibiotic daptomycin and its congeners taromycins discovered using a metagenomics approach; differences in structure are highlighted in red.

Nonribosomal peptides are synthesized by nonribosomal peptide synthases, which are easily distinguishable in genome data. These complex protein complexes provide a great deal of structural information for bioinformatics analysis and draw significant attention as targets for genetic manipulations. Significant success was achieved in the search for nonribosomal peptides with antibiotic activity. The lipopeptide taromycin (Figure 11) was the first compound obtained via transformation-associated recombination (TAR) cloning from marine actinomycete *Saccharomonospora* sp. CNQ-490 into the model organism *Streptomyces coelicolor* [100,101,102].

**Figure 12:**
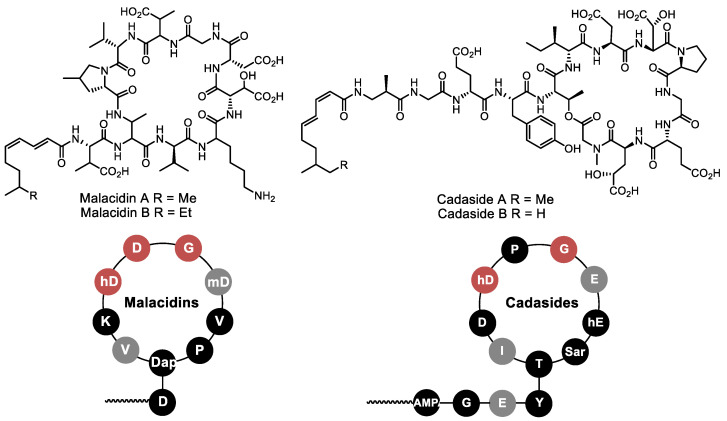
Lipopeptide antibiotics malacidins and cadasides discovered using metagenomics search for the Ca-binding motif. In the lower diagram: Red—conserved Ca-binding motif, gray—D-amino acids.

Subsequently, this technique was also applied to other nonribosomal peptides. The group of Prof. S. Brady (Rockefeller University) used the sequence responsible for the inclusion of the Asp-X-Asp-Gly motif as a key idea in the metagenomic search. This fragment is responsible for the binding of calcium ions in various calcium-dependent cyclo(depsi)peptides, such as daptomycin. Successful gene transfer using the TAR platform led to isolation of the original calcium-dependent peptide antibiotic malacidin (Figure 12) [103]. This approach was further developed with cadazides—they also show a highly distorted calcium-binding motif [104]. A similar method was used to search for menaquinone (MK)-binding antibiotics; the conserved sequence GXLXXXW, which represents the minimal MK-binding motif, was used for the genomic search. This approach led to the identification of six new structurally distinct MK-binding peptides [105].

**Figure 13:**
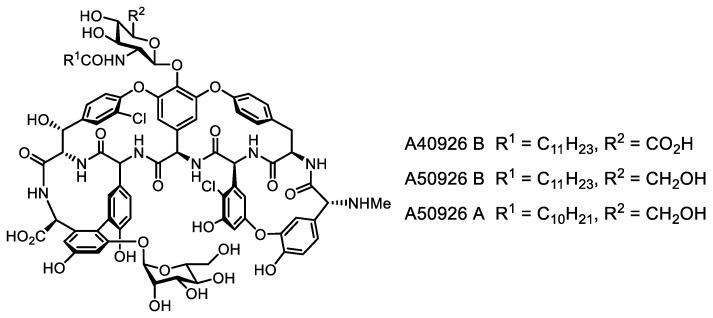
Glycopeptide antibiotics A50926 and A40926.

The study of clusters homologous to known BGC of peptide antibiotics remains a promising approach to finding new compounds. A new glycopeptide antibiotic A50926, close to A40926 known as the precursor of the semi-synthetic glycopeptide dalbavancin [106], was discovered this way (Figure 13).

**Figure 14:**
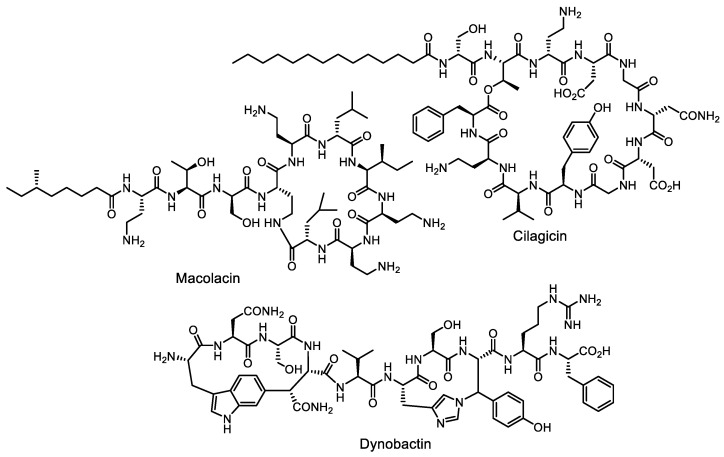
New antimicrobial (lipo)peptide antibiotics macolacin, cilagicin and dynobactin, discovered by genome mining.

The study of homologous clusters was also effective in finding colistin-resistant peptide antibiotics, and genome mining of bacterial genomes for polymyxin-like BGCs revealed macolacin (Figure 14), a structurally divergent colistin congener active against co-li-stin-resistant strains [107]. The structure predicted from genomic data was obtained by chemical synthesis. The lipopeptide cilagicin (Figure 14), which inhibits cell wall biosynthesis of Gram-positive bacteria by an unusual mechanism, was synthesized the same way [108]. Another successful example of a genomic search by cluster homology is the discovery of dynobactin (Figure 14), which is structurally very different from darobactin, but also acts via BamA [109].

The NRPminer platform was developed to search for clusters of non-ribosomal peptides, which made it possible to identify several new families of non-ribosomal peptides by integrating genomic and metabolomic datasets [110].

The genome mining strategy has proven very promising for finding new RiPPs [111]. For example, novel head-to-tail cyclized bacteriocins have been identified using a transporter protein-based genome mining strategy [112]. Approaches to identifying bacteriocin clusters continue to evolve, with BADASS recently proposed for searching for bacteriocin clusters in metagenomic data [113]. In recent years, a series of bacteriocin clusters have been identified, and new peptides, such as sactipeptide estercticin A, have been isolated [114].

Among polyketides, examples of successful genomic approaches have been rarer in recent years than for peptide antibiotics. The discovery of a series of natural macrolactams [115] using genomic signature-based PCR screening of a bacterial DNA library can be noted. PCR-based screening was used to search for glycosylated streptomycete metabolites. Strains associated with leaf-cutting ants were examined for the presence of deoxysugar biosynthesis gene clusters [116], and several novel macrolactams and angucyclines were found as a result. For aromatic polyketides synthesized by type II polyketide synthases, new productive approaches for bioinformatic analysis of genomic data were developed [117].

**Figure 15:**
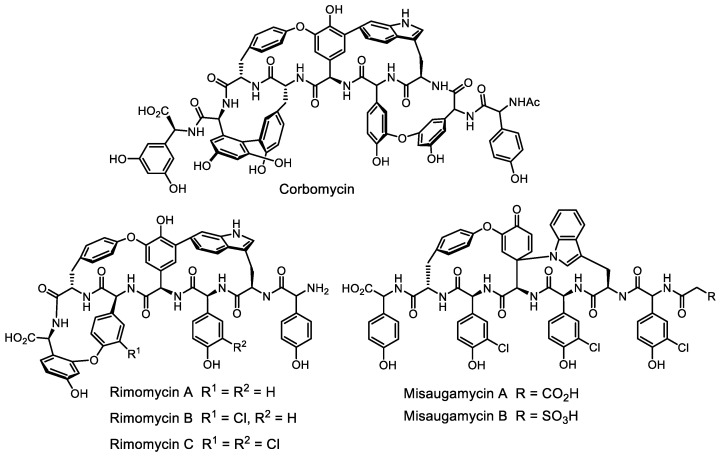
Structures of corbomycin, rimomycins A–C, and misaugamycins A,B.

Several mining strategies are independent of the natural product class or biosynthetic enzyme homologies and, therefore, can potentially access cryptic BGCs for ‘true’ chemical novelty [118]. An approach to the problem of gene cluster selection by the group of Prof. G. Wright (McMaster University) proposes focusing on the genetic determinants of resistance—in the producer or the test culture [119,120]. This method was effective in the search for nonribosomal peptides: corbomycin (Figure 15), a new peptide with an original mechanism of action, was isolated this way [121]. The corbomycin product was selected based on the fact that the autoresistance gene located in the biosynthesis cluster is very different from the familiar glycopeptides *vanHA* and *vanY*. As a continuation of this work, five new corbomycin family members of high structural novelty, rimomycin-A/B/C and misaugamycin-A/B peptides, were isolated (Figure 15) [122].

A review of earlier work using this methodology (self-resistance-directed natural product discovery) is presented by Prof. Yi Tang and colleagues [123]. A specialized platform for the search for such resistance determinants (Antibiotic-Resistant Target Seeker (ARTS)) [124,125] and a database with pre-computed ARTS results for >70,000 genomes were developed by Prof. Ziemert’s group [126]. This approach, called Ψ-footprinting, was also recently adapted to the search for protein synthesis inhibitors (PSI = Ψ) [127].

**Figure 16:**
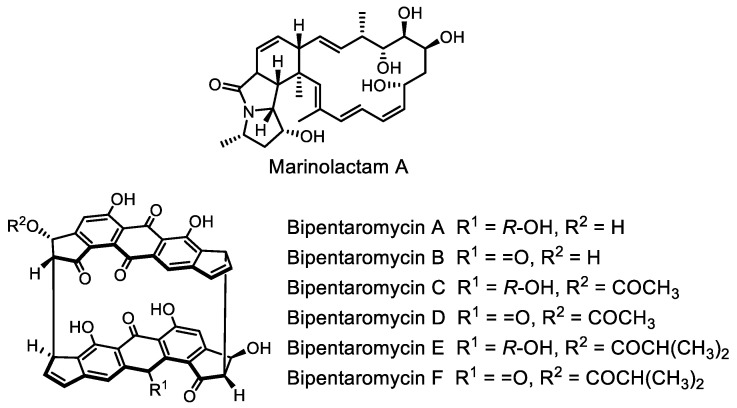
Structures of marinolactam A and bipentaromycins A–F.

However, direct cloning of large BGCs remains challenging. We note several works that expand our capabilities in this direction. An efficient in vitro platform for directly capturing large BGCs, named CAT-FISHING (CRISPR/Cas12a-mediated fast direct biosynthetic gene cluster cloning), was developed recently [128]. As a proof-of-concept, several large BGCs from various actinomycetal genomic DNA samples were efficiently captured by CAT-FISHING, the largest of which was 145 kb with 75% GC content. A new macrolactam compound with anticancer activity, marinolactam A (Figure 16), was isolated using this method. A previous achievement was made using the CAPTURE system [129], which enables the induction of the biosynthesis of antimicrobial polyketides bipentaromycins A–F (Figure 16).

In general, the described successful examples of the use of genomic approaches show the prospects of this direction; however, unfortunately, in practice, the implementation of such an approach at all stages is associated with serious difficulties [130]. First, the principles of analysis of genetic information to identify metabolites that are of interest for their biological properties are not completely clear. In addition, difficulties arise in obtaining the metabolites revealed by genomic studies. Linking genes to compounds remains a challenging part of the workflow [118]. Recent promising approaches to solving this problem include the development of IsoAnalyst—an isotopic labelling approach [131]—and the development of the hcapca (Automated Hierarchical Clustering and Principal Component Analysis) methods [132,133]. The gene clusters of interest often have a complex organizaton and are large in size (>50 kb) and integrated into the progenitor development cycle via intricate signaling cascades. Nevertheless, the huge number of publications in recent years and numerous discoveries of natural metabolites with a high degree of chemical novelty and unusual mechanisms of action indicate that genome-driven approaches are among the most promising in the field of natural product discovery.

### 3.2. Biosynthetic Gene Cluster Activation

Here we grouped together various methods that are based on the addition of a small amount of an “inductor”—a small molecule, a biopolymer, or a fragment of an inactivated cell. Interestingly, in some cases, other antibiotics, e.g., produced by another species of actinobacteria, can serve as an effective inducer. This greatly enriches and complicates our understanding of what antibiotics are for microorganisms [134,135,136,137,138].

**Figure 17:**
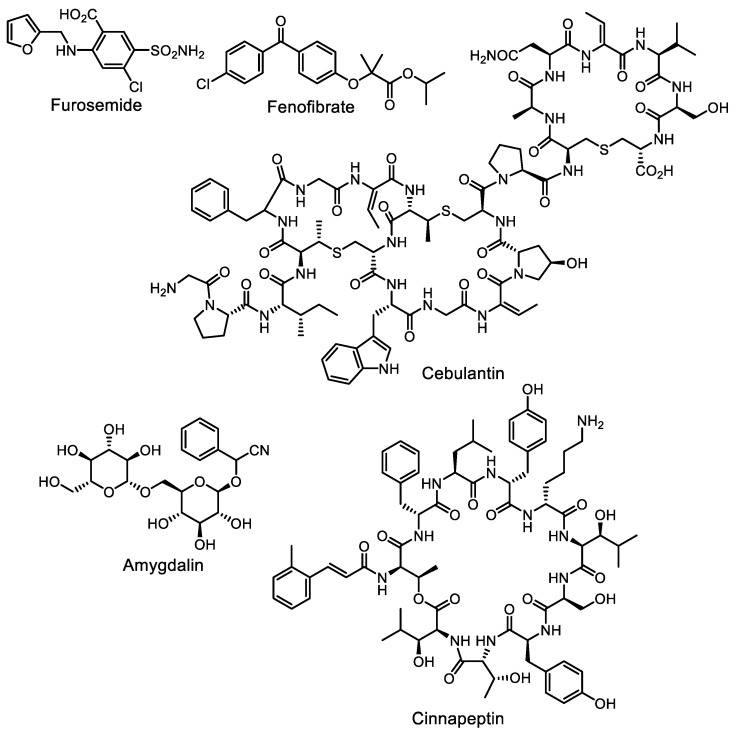
Peptide antibiotic cebulantin and its inductors furosemide and fenofibrate; peptide antibiotic cinnapeptin and its inductor amygdalin.

The group of Prof. M. Seyedsayamdost is especially active in this area. They are trying to use high-throughput screening methods to find small-molecule inductors (or “elicitors”). Specialized software was developed to analyze the datasets obtained from a large number of elicitors (Metabolomics Explorer, or MetEx, https://mo.princeton.edu/MetEx/ (accessed on 20 April 2023)) [139]. Recent successes include the following works. Cebulantin, an antibiotic exhibiting moderate activity against Gram-negative bacteria, especially of genus *Vibrio*, is produced by the rare actinomycete *Saccharopolyspora cebuensis* when one of the inducers, furosemide or fenofibrate, is added to the medium (Figure 17) [140]. Another cyclopeptide antibiotic, cinnapeptin, was found to result from the action of the plant glycoside amygdalin (Figure 17) on a *Streptomyces ghanaensis* culture [141].

**Figure 18:**
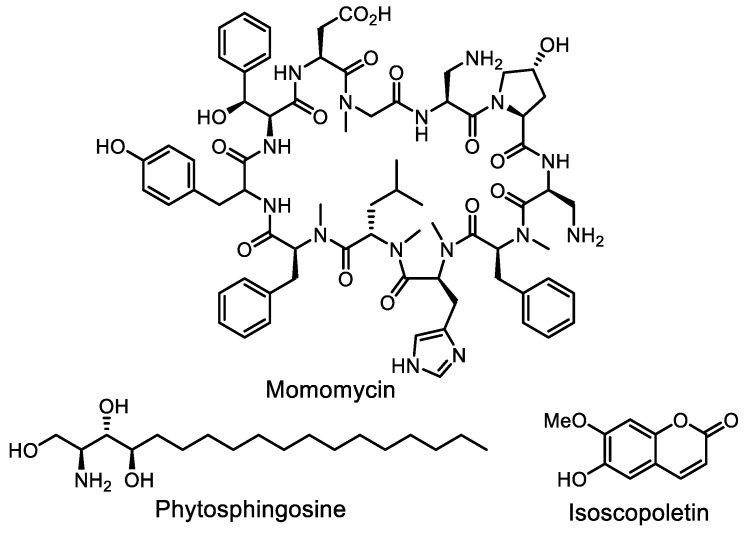
Cyclopeptide momomycin and its elicitors.

Interestingly, the new cytotoxic peptide antibiotic momomycin was discovered in a culture of *Streptomyces rimosus* ATCC 10970, a well-known industrial oxytetracycline producer [142]. The biosynthesis of momomycin is enabled by plant metabolites phytosphingosine and isoscopoletin (Figure 18). A targeted search for antiproliferative compounds using elicitation techniques has been described [143].

Despite a large number of interesting results, there is no pattern among the inducers used: Both natural substances of very different origins and synthetic ones turned out to be inducers.

**Figure 19:**
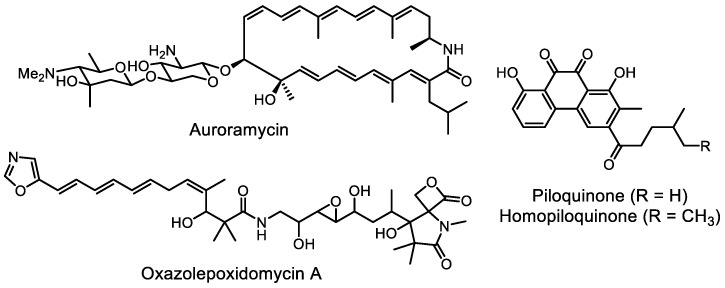
Structures of auroramycin, oxazolepoxidomycin A, piloquinone and homopiloquinone.

More selective methods of BGCs activation do not require screening a wide panel of activating factors. Initially, hopes were pinned on genetic engineering manipulations with regulatory (global or pathway-specific) genes and promoters. Early successes in the activation of antibiotic biosynthesis using this strategy are summarized in previous reviews [144,145,146]. Some of the most recent works are CRISPR/Cas-based strategies for unearthing the hidden chemical space [147]. Using a CRISPR-Cas9 gene cluster activation strategy, a unique macrolactam glycosylated by two aminosugars, auroramycin (Figure 19), was isolated [148,149]. A transcription factor decoy strategy for targeted activation of large BGCs was reported [150]. Transcription factor decoys are DNA molecules designed to interfere with gene regulation by mimicking regulatory DNAs that are bound to regulators and thus prevent the latter from binding to their cognate DNA targets. This could result in the de-repression of a target silent BGC as well as the de-activation of a target naturally active BGC. Based on this approach, a new oxazole compound (Figure 19) was identified.

Ribosomal engineering as an approach was formulated and actively developed by a group of Japanese researchers under the leadership of Prof. Ochi [151,152]. Its essence is the selection of mutants of the producing strain on a media with increasing concentrations of the corresponding antibiotic translation inhibitor. The accumulation of mutations changing the structure and normal functioning of the ribosomal machinery leads to a qualitative change in the metabolome. Ribosomal engineering is still often used today as an approach to strain improvement [153].

A new approach to ribosomal engineering—so-called “Transcription–Translation in One” (TTO)—was recently described [154]. This approach aims to alter the metabolite profiles of the target strains by directly overexpressing exogenous rpsL (encoding ribosomal protein S12) and rpoB (encoding the RNA polymerase β subunit) genes containing mutations for biosynthesis activation using a plug-and-play plasmid system. TTO was successfully applied to activating cryptic BGCs in three *Streptomyces* strains: New polyketide antibiotics, piloquinone and homopiloquinone (Figure 19), were discovered.

**Figure 20:**
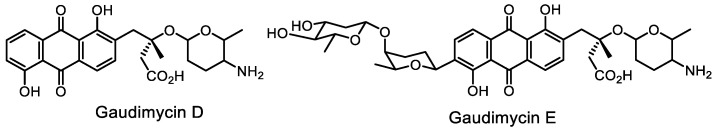
Structures of gaudimycins D, E discovered by means of RGMS.

Reporter-guided mutant selection (RGMS) was developed as an effective and widely applicable method for targeted activation of silent BGCs. RGMS combines two technologies: genome-scale random mutagenesis to generate genetic diversity and a promoter–reporter system to facilitate the selection of mutants in which transcription from the targeted gene cluster was activated [155]. The strategy was applied to the *pga* gene cluster in *Streptomyces* sp. PGA64, leading to the identification of two new anthraquinone aminoglycosides, gaudimycin D and E (Figure 20). Using improved RGMS, several cryptic metabolites from mutant libraries of various *Burkholderia* species were identified. The authors used transposon mutagenesis instead of UV [156] and MS-based metabolomics instead of a reporter construct [157,158].

### 3.3. Reporter Strains and Mechanism-Guided Isolation

**Figure 21:**
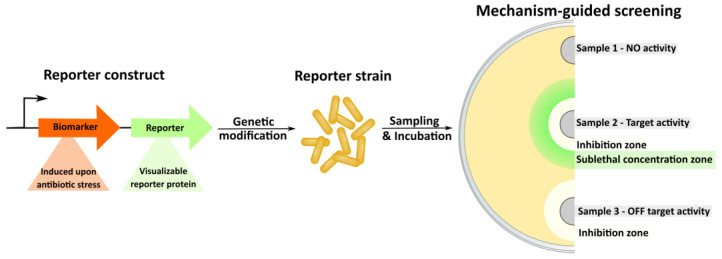
Reporter strain-assisted screening strategy.

Establishing the molecular target and mechanism of action of an antibiotic is important not only in terms of research but also for further rational modification and evaluation of the potential of the compound. Today, this information is also needed to promote a potential drug to the pharmaceutical market [159]. Typically, additional profiling and biochemical tests need to be performed for a new natural product to elucidate its mechanism of action (MoA). Among various assays for target determination, reporter strains are most suitable for mechanism-based screening and antibiotic discovery [160]. The effect of reporter strains is based on a selective increase in the expression of a gene under the action of sublethal concentrations of the antibiotic. There are many such systems that allow a bacterial cell to activate genes, whose products neutralize or mitigate the effects of antibacterial compounds. For easy visualization, the activated gene product needs to be replaced with a reporter construct (Figure 21) [161].

A test culture or a small panel of strains that allows immediate identification of the active substance and its target/mechanism of action is a highly productive approach to screening. The basic approaches to reporter strains and their applications in antibiotic screening have been summarized previously [161]. Further developments and successful applications in this field are rather scarce in the past few years.

Double fluorescent reporter strains for high-throughput screening [162] were shown to be effective for mechanism-based sorting of antimicrobial compounds: the reporter strain, based on a susceptible mutant of *E. coli* as the model organism, sorts out antimicrobials that cause ribosome stalling and those that induce the SOS response due to DNA damage. This approach was very fruitful primarily for MoA clarification of known compounds. For example, it was recently found that the aromatic polyketide antibiotic tetracenomycin X is a potent inhibitor of protein synthesis and does not induce DNA damage as previously thought [163]. Recently, a pipeline based on the dual reporter system was upgraded for utilization in citizen science projects [164] by the introduction of reporter genes visualized by the naked eye.

The most recent advance in this field is the reporter strain panel based on *Bacillus subtilis* as the model organism [165]. The bioreporters demonstrate visible promoter induction under various conditions: cell envelope stress, lipid II cycle stress, DNA stress, RNA stress and translation arrest. Therefore, testing against the bioreporter panel provides valuable insight into the most common types of antimicrobial MoA of the tested compounds. The panel was validated on known antibiotics and applied to the screening of 500 strains, and the signals of the bioreporters matched the described MoA of known and dereplicated antibiotics in all cases.

Targeted mechanism-guided search and identification of new scaffolds could be based on chemical genetics. This emerging approach was recently validated in anti-tubercular activity screening [166]. New chemotypes and new promising targets were established, although for synthetic compounds. In a similar vein, but on a smaller scale, natural products have been screened for their potential effect on bacterial biotin biosynthesis. A known biotin antimetabolite, amiclenomycin, was isolated, and its cellular target—the biotin transporter yigM—was identified simultaneously [167]. Early identification of chemical–gene interactions could open a new pathway for antibiotic discovery [168].

To conclude, reporter strains have drawn significantly less attention in the past few years than genome-guided approaches (described in Section 3.1). Although MoA-based screening is a rather questionable strategy for the development of novel antibiotics with valuable therapeutic properties, these methods are still very promising for the development of novel targets. As the main result, reporter strain-based approaches lead to a profound understanding of molecular modes of action for known antibiotics (including secondary mechanisms), thus enabling further rational structural design.

## 4. Chemistry

**Figure 22:**
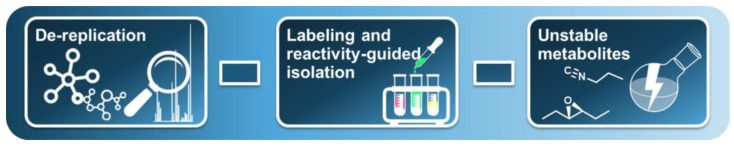
Basic chemical approaches to the search for new antibiotics.

Although we do not consider advances in the field of synthetic compounds and semi-synthetic modification of natural products in this review, chemistry still remains one of the most important areas of innovation in the search for new natural compounds (Figure 22). First of all, effective and widely used approaches for identifying and prioritizing natural antibiotics are based on chemical methods—the first section is devoted to an overview of this area. However, the experience in highly selective transformations accumulated within the framework of bioorthogonal chemistry makes it possible today to use some reagents for screening and prioritization of certain structural groups directly in extracts and mixtures of natural origin (see Section 4.2). The last section is devoted to methods for working with labile compounds.

### 4.1. Dereplication

The term “dereplication” was used in the first CRC Handbook of Antibiotic Compounds, published in 1980, to denote the recognition and elimination of already-studied active substances at the early stages of the screening process [169,170]. Now, it can be used in a broader sense as a technology for the detection and/or elimination of repeating samples (cultures, compound mixtures or pure compounds) or samples containing well-known active compounds in natural product screening. In this review, “dereplication” is defined as an analytical technique or a complex approach which enables the detection of known compounds at the first step of antibiotic screening: in culture broths, extracts and crude fractionated mixtures. As a result, dereplication is a solution to the re-discovery problem and allows us to prioritize the objects of study, focusing the resources only on new cultures and compounds.

**Figure 23:**
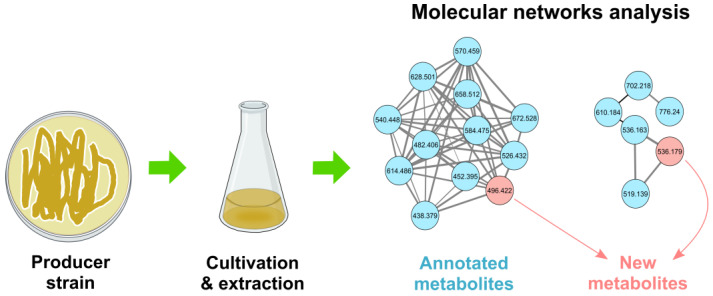
Molecular networking used for dereplication.

From early reviews [169,171], most works in the area relate to MS-based dereplication. Mass spectrometry is a powerful and informative technique for this use thanks to its high sensitivity, valuable structural information and reproducibility. The current standard in MS dereplication (Figure 23) is based on public (or partially public, such as DNP) databases, e.g., the Global Natural Products Social molecular network (GNPS, https://gnps.ucsd.edu/ accessed on 20 April 2023) [172], the Natural Products Atlas (NPAtlas, https://www.npatlas.org/ accessed on 20 April 2023) [173,174], and the Dictionary of Natural Products (DNP, http://dnp.chemnetbase.com/ accessed on 20 April 2023). Other specific databases and the main problems with their use were summarized in recent reviews [175,176].

Another option based on MS/MS fragmentation is molecular networking (MN). MN implemented in GNPS could be useful for the study of complex mixtures. The hcapca (hierarchical cluster analysis with principal component analysis) algorithm can identify similar patterns of fragmentation and unique and ubiquitous components and simplify the search for congeners or characteristic compounds [177,178]. MN is a very promising approach for a more complete and in-depth analysis of LC-MS/MS data routinely used for dereplication.

With the help of MN, 18 previously unknown cyclosporins (cyclopeptides with immunosuppressive activity) [179], four novel valinomycin congeners [180] and new ribosome-targeting antibiotics hetiamacins E and F [181] were discovered. Now, MN is becoming a key method for visualizing and annotating the chemical space in untargeted metabolomics [182,183,184,185].

Dereplication based on NMR spectral data is under active development. We should mention several works:

- The DEREP-NP (https://github.com/clzani/DEREP-NP accessed on 20 April 2023) platform has been developed for structural feature search in the UNPD public NMR database [186]. Later, diffusion-ordered NMR spectroscopy (DOSY)-related functionality was implemented [187].
- To decipher complex mixtures using ^13^C-NMR data, MixONat (https://sourceforge.net/projects/mixonat/ accessed on 20 April 2023) open-source software was developed [188].
- The MADByTE data analysis platform (Metabolomics and Dereplication by Two-Dimensional Experiments, https://github.com/liningtonlab/MADByTE accessed on 20 April 2023) for complex mixture analysis was developed. This platform employs a combination of TOCSY and HSQC spectra to identify spin system features within complex mixtures and create a chemical similarity network [189].

This methodology has several significant limitations:

- Poor compatibility with the main methods of mixture separation: LC-NMR is an exotic combination, unlike LC-MS.
- Limited throughput due to the significant duration of registration of the spectra.
- Distinguishing the components of complex mixtures is difficult: the characteristic spectral range for natural compounds (0–10 ppm for ^1^H signals) is very narrow and it takes time to register reliable signal at a sufficient resolution in mixtures with additional correlations and/or additional computation [190,191].

The third type of data available for efficient dereplication and prioritization of natural antibiotics is activity fingerprinting. The bioactivity fingerprint consists of data on the activity of a given sample in various cultures. To search for new antibiotics, it is useful to measure inhibitory concentrations against a representative panel of microorganisms. Obviously, for identical substances (or substances with an identical mechanism of action), similar patterns will be observed in their bioactivity fingerprints.

**Figure 24:**
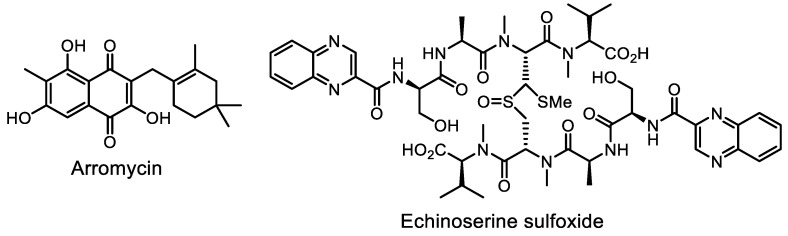
Structures of arromycin and echinoserine sulfoxide.

Historically, the first platform based on this strategy was BioMAP (antibiotic mode of action profile) [192]. Using a panel of clinically relevant bacterial strains, the presence of known antibiotics in natural product extracts was accurately predicted, and arromycin (Figure 24)—a naphthoquinone-based antibiotic from the marine natural product library—was discovered.

A similar approach was used with a library of antibiotic-resistant transformants of both wild-type *E. coli* BW25113 and a hyperpermeable, efflux-deficient mutant of *E. coli* BW25113, Δ*bamA*Δ*tolC* [193]. Screening using the antibiotic resistance platform (ARP) was carried out, and a new echinoserine congener was isolated.

**Figure 25:**
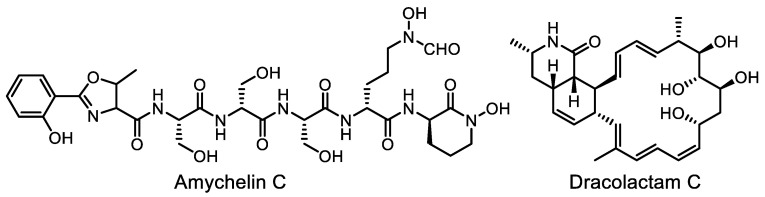
Structures of amychelin C and dracolactam C.

A new platform, NPAnalyst (www.npanalyst.org, accessed on 20 April 2023), has been developed for direct prediction of metabolite bioactivity profiles from complex mixtures. This platform is compatible with both mzML (mass spectrometry open-data format) and most open-data processing platforms (GNPS and MZmine 2). Validation of the platform was performed by analyzing a “low-resolution” antimicrobial bioassay dataset for 925 natural product prefractions. Two new antibiotics, dracolactam C and amychelin C (Figure 25), were described [194].

### 4.2. Chemical Labeling and Reactivity-Guided Isolation

Chemical derivatization techniques are well-known and are used extensively for routine analysis. However, only recently they were adopted as a platform for natural product screening. The main idea is based on selective reagents capable of labeling the functional groups or structural features of natural products right in the extracts or pre-fractioned mixtures. Despite the original metabolites becoming modified, the resulting adducts often have enhanced visibility by UV and MS. This emerging topic in natural product chemistry was recently described [195].

**Figure 26:**
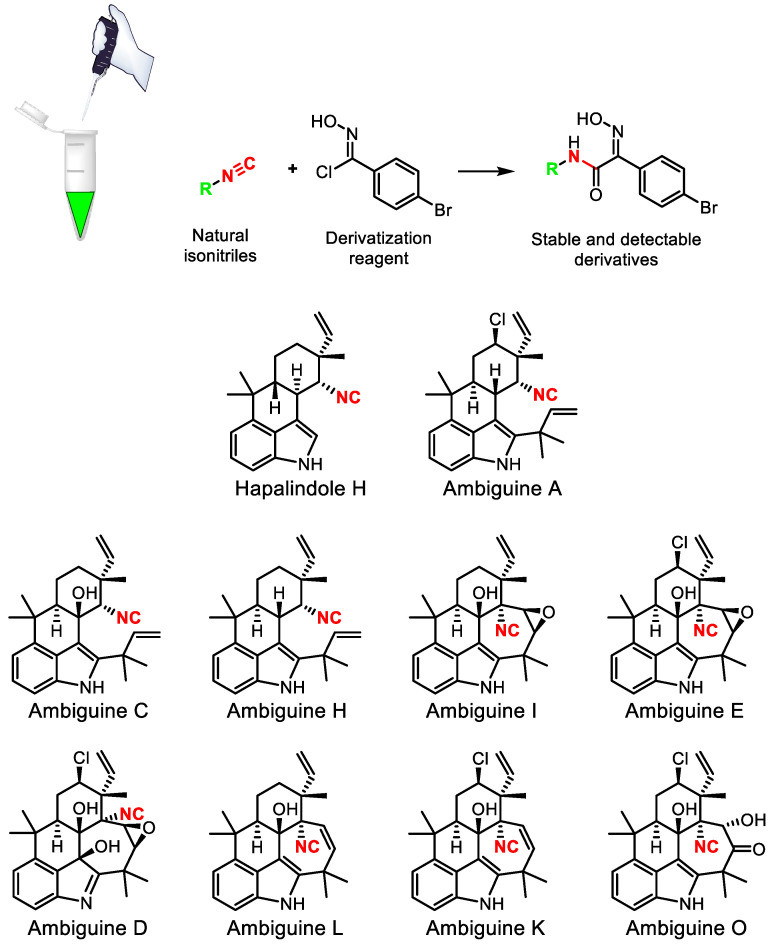
Chemical derivatization of labile isonitrile natural products.

As an illustration of the approach, we could note a recent work [196] on reactivity-based screening for the detection and isolation of alkaloid and terpene isonitriles in the cyanobacterium *Fischerella ambigua* and a marine sponge of the order Bubarida (Figure 26).

The modification of the antibiotic can be reversible. For example, a reversible modification of amines was recently reported [197].

### 4.3. Methods for Detection and Isolation of Unstable Metabolites

The first natural antibiotic, penicillin, was elusive for researchers for many years due to its thermolabile nature. Only the development of freeze drying could solve the problem and enable its research and further medical use. Perhaps the development of new methods for working with labile substances will open up new opportunities and new chemotypes in the field of natural antibiotics.

The isolation of light-sensitive, highly volatile, chemically active substances from complex mixtures is still challenging, and no specific techniques address this problem. The main advantages and relevant problems in the field are summarized in a review [198].

**Figure 27:**
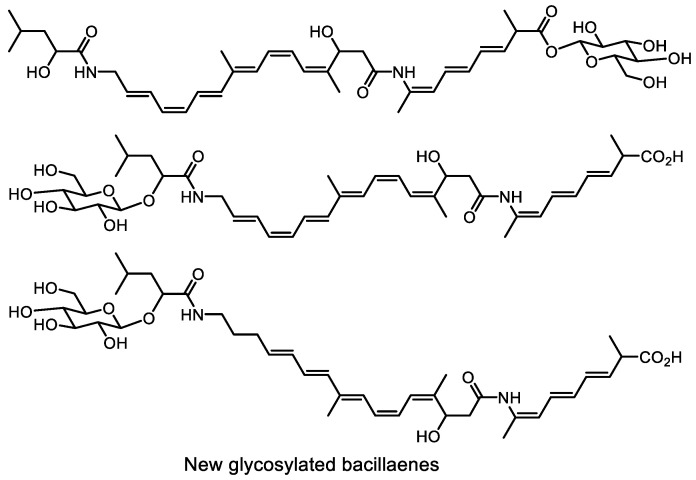
Structures of unstable bacillaenes.

Some progress was achieved with bacillaenes—well-known light- and oxygen-sensitive unsaturated compounds. New bacillaene structures (Figure 27) were identified in compound mixtures using the DANS-SVI (differential analysis of 2D NMR spectrum−single spectrum with variable intensities) method. After identification, compounds of interest were isolated under strictly controlled conditions [199].

## 5. Conclusions and Outlook

**Figure 28:**
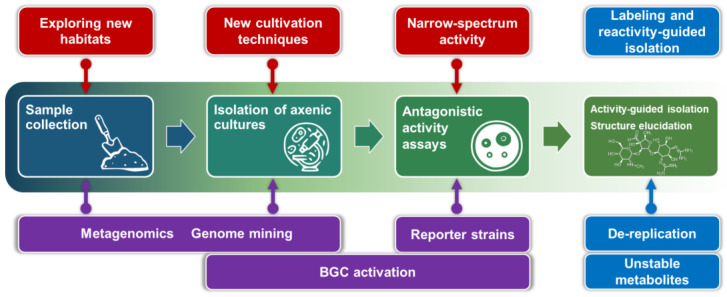
Schematic representation of modern trends in antibiotic discovery.

The spread of antibiotic resistant-strains makes the development of novel antimicrobials outstandingly important. Nonetheless, effective and efficient development of new drugs is impossible without the discovery of appropriate drug leads. In this account, we summarized current problems in natural antibiotic discovery and the main approaches to solving them. None of these approaches provide a conclusive answer to the question ‘How do we make antibiotics great again?’. Moreover, the basic workflow remains the same and generally resembles traditional phenotypic screening (the “Waksman platform”) (Figure 28).

All of the described methods are improvements of some stage in the classic discovery model. The integration of these improvements could lead to a significant increase in the discovery rate of novel antibiotics. Combining various datasets (e.g., genomic and metabolomics) was shown to provide novel types of valuable information. Nonetheless, further integration of the data requires advanced instrumentation, technologies and computational resources.

## Data Availability

Not applicable.

## Abbreviations

BGC, biosynthetic gene cluster; CRISPR, clustered regularly interspaced short palindromic repeats; DNA, deoxyribonucleic acid; iChip, isolation chip technology; FACS, fluorescence-activated cell sorting; GNPS, Global Natural Products Social molecular network; HSQC, heteronuclear single quantum coherence spectroscopy; LC, liquid chromatography; MDE, microfluidic double water-in-oil-in-water emulsion; MoA, mechanism of action; MN, molecular networking; MS, mass-spectra(l); NMR, nuclear magnetic resonance; NRP, nonribosomal peptide; PCR, polymerase chain reaction; RGMS, reporter-guided mutant selection; RiPP, ribosomally synthesized and post-translationally modified peptide; RNA, ribonucleic acid; TAR, transformation-associated recombination; TOCSY, total correlation spectroscopy; UV, ultraviolet; WHO, World Health Organisation.
